# The SV40 Late Protein VP4 Is a Viroporin that Forms Pores to Disrupt Membranes for Viral Release

**DOI:** 10.1371/journal.ppat.1002116

**Published:** 2011-06-30

**Authors:** Smita Raghava, Kristina M. Giorda, Fabian B. Romano, Alejandro P. Heuck, Daniel N. Hebert

**Affiliations:** Department of Biochemistry and Molecular Biology, Program in Molecular and Cellular Biology, University of Massachusetts, Amherst, Massachusetts, United States of America; Cornell University, United States of America

## Abstract

Nonenveloped viruses are generally released by the timely lysis of the host cell by a poorly understood process. For the nonenveloped virus SV40, virions assemble in the nucleus and then must be released from the host cell without being encapsulated by cellular membranes. This process appears to involve the well-controlled insertion of viral proteins into host cellular membranes rendering them permeable to large molecules. VP4 is a newly identified SV40 gene product that is expressed at late times during the viral life cycle that corresponds to the time of cell lysis. To investigate the role of this late expressed protein in viral release, water-soluble VP4 was expressed and purified as a GST fusion protein from bacteria. Purified VP4 was found to efficiently bind biological membranes and support their disruption. VP4 perforated membranes by directly interacting with the membrane bilayer as demonstrated by flotation assays and the release of fluorescent markers encapsulated into large unilamellar vesicles or liposomes. The central hydrophobic domain of VP4 was essential for membrane binding and disruption. VP4 displayed a preference for membranes comprised of lipids that replicated the composition of the plasma membranes over that of nuclear membranes. Phosphatidylethanolamine, a lipid found at high levels in bacterial membranes, was inhibitory against the membrane perforation activity of VP4. The disruption of membranes by VP4 involved the formation of pores of ∼3 nm inner diameter in mammalian cells including permissive SV40 host cells. Altogether, these results support a central role of VP4 acting as a viroporin in the perforation of cellular membranes to trigger SV40 viral release.

## Author Summary

Viruses exploit host cells for their propagation. Once an adequate number of viral particles have been assembled within the cell through the aid of cellular machinery of the host cell, the virus must be released from the cell for the virus to spread. For nonenveloped viruses or viruses that are solely encapsulated by a protein shell, this step most commonly involves the perforation of cellular membranes resulting in the lysis or death of the host cell. The mechanism for how this key terminal step in the viral life cycle is performed is poorly understood. We demonstrated that for the model nonenveloped virus SV40, the newly discovered virally encoded protein, termed VP4, perforates membranes by forming pores with a diameter of ∼3 nm in host cell membranes. While these pores are not of a sufficient size to provide a conduit that permits the movement of the virus through the membrane, they support membrane destabilization that leads to the disintegration of the membrane of the host cell and viral release.

## Introduction

The virus life cycle is comprised of a number of consecutive, discrete, and tightly regulated steps. These steps include binding, internalization, penetration, replication, assembly, and release. The final step of the viral life cycle involves the exit of viral progeny from the cell to support virus dissemination for infection. Most enveloped virions are released from the cell by a budding or a membrane fission reaction with the virus acquiring a membrane coat during this process [1], [2]. Nonenveloped viral release generally requires cell lysis after the viral multiplication cycle has been completed [3], [4]. While this is a critical fundamental event in the viral life cycle, the mechanism of nonenveloped viral release is weakly defined.

The timing of the nonenveloped virus exit step is critical for an optimal life cycle, as lysis should occur immediately after an adequate number of virus particles have been assembled. This lytic event is associated with the disruption of cellular membranes, which leads to cell death. However, a cytolytic pathway that avoids apoptosis would also be advantageous, as this would ensure that the virus is not encapsulated by apoptotic membrane blebs, which would inhibit binding to host cell membranes.

**Figure 1:**
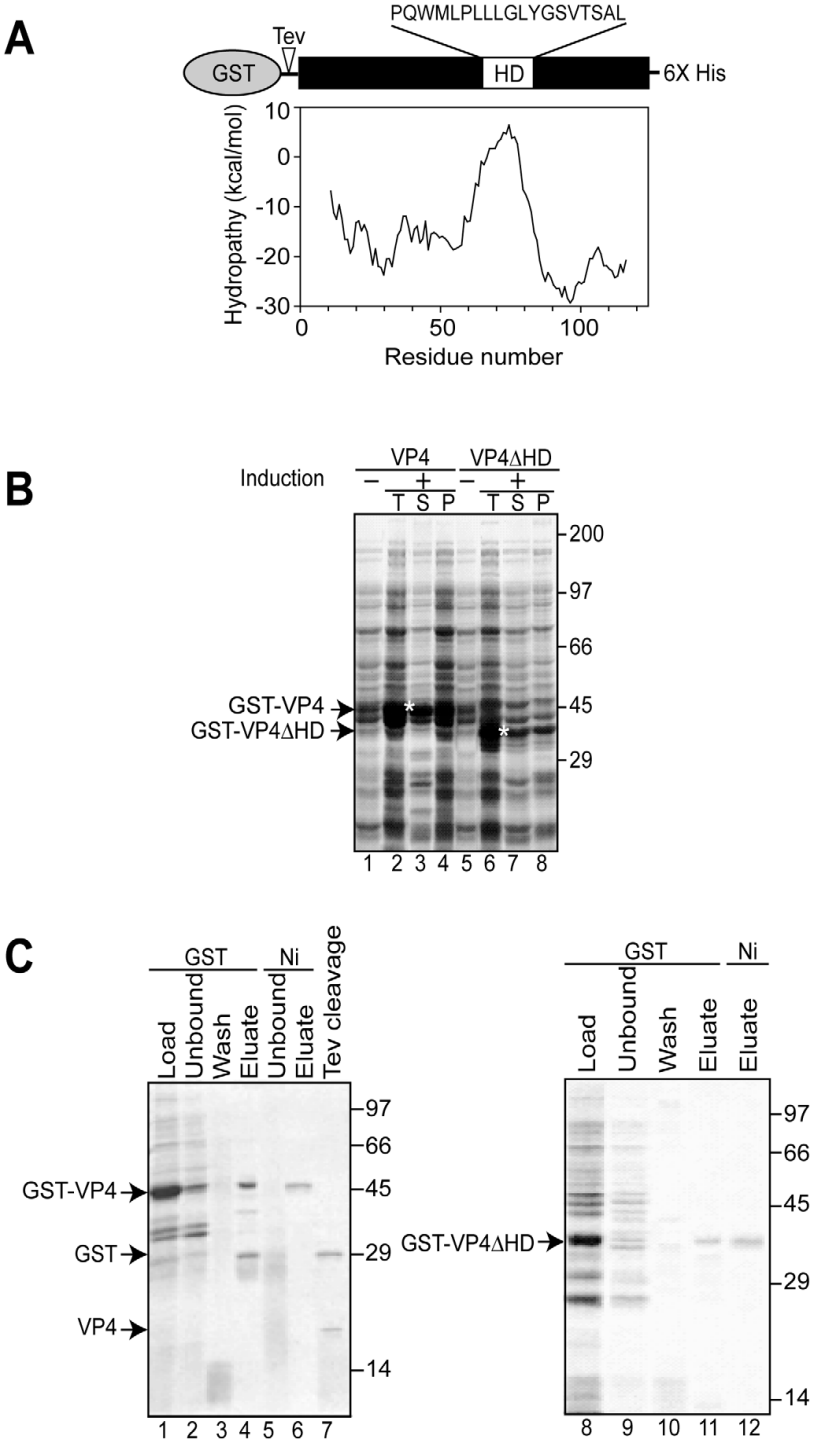
Bacterial expression and purification of VP4. (A) Schematic representation of the VP4 construct containing N-terminal GST and C-terminal 6xHis tags. VP4 contains a hydrophobic domain (HD) with sequence designated. The hydrophobicity plot of VP4 using Membrane Protein Explorer version 3 [46] is displayed. (B) Influence of the osmolyte, proline, on the solubility of GST-VP4 and GST-VP4ΔHD expressed in bacteria before (−) and after (+) induction with IPTG. Total (T), supernatant (S), pellet (P) fractions were resolved by SDS-PAGE and visualized by coomassie brilliant blue staining. Soluble GST-VP4 and GST-VP4ΔHD are designated by asterisks. (C) SDS-PAGE analysis of two-step affinity purification of GST-VP4 and GST-VP4ΔHD.

Simian Vacuolating virus 40 (SV40) is a member of the polyomavirus family. SV40 was the first eukaryotic virus sequenced over thirty years ago [5], [6] and studies of SV40 have advanced our understanding of nuclear transport, transcriptional regulation, and cell transformation [7]. Therefore, it serves as an excellent paradigmatic virus to help expand our knowledge of the undefined stages of the nonenveloped viral life cycle including viral release. Three structural late proteins are found in the viral particle: VP1, VP2 and VP3. VP1 forms 72-pentameric capsomeres to create the viral capsid. Each capsomere contains a single copy of either minor structural protein VP2 or VP3 in its central cavity [8]. The same transcript encodes VP2 and VP3 with translation initiation occurring from sequential Met residues to create N-terminal truncations of each other. We recently discovered that another downstream Met in the VP2/VP3 transcript also acted as a translation initiation site to encode an additional protein termed VP4 [9]. VP4 is a 125 amino acid protein with a central hydrophobic domain (Figure 1A). As the synthesis of VP4 coincided with the time of viral-mediated cell lysis, VP4 was proposed to play a role in viral release.

In this study, we have investigated the ability of VP4 to bind and disrupt lipid membranes. For this purpose, a tagged version of VP4 was constructed and its properties of membrane binding and disruption were analyzed. These studies showed that VP4 efficiently bound red blood cell (RBC) membranes and supported membrane perforation as probed with a hemolysis assay. In addition, VP4 also disrupted liposomes mimicking the composition of mammalian plasma and nuclear membranes. Deleting or substituting the hydrophobic domain of VP4 drastically abolished its membrane disruptive activity, indicative of a role for the hydrophobic domain in membrane lysis. VP4 formed small ∼3 nm diameter pores in mammalian membranes. Altogether, these results support a central role for VP4 in the direct disruption of cellular membranes to trigger SV40 viral release.

## Results

### Bacterial expression and purification of VP4

To obtain recombinant SV40 VP4 for analysis of its activity, initially VP4 containing a C-terminal His tag was expressed in *Escherichia coli*. His tagged VP4 was undetected after 12 hr of induction (data not shown). The SV40 late protein VP4 contains a 19 amino acid central hydrophobic domain as suggested by hydrophobicity analysis (Figure 1A). The hydrophobic nature of VP4 combined with its potential lytic activity pose challenges for its stable bacterial expression, and its purification as a water-soluble and active protein [9].

The fusion of water-soluble proteins to the N-terminus of aggregation-prone polypeptides is an efficient and effective approach to maintain the solubility of a protein suitable for bacterial expression [10]. The addition of a bulky soluble protein such as glutathione S-transferase (GST) might diminish potential lethal properties of VP4 that contribute to its inability to be expressed in *E. coli*. To test if this strategy would support bacterial VP4 expression, a construct of VP4 with an N-terminal GST tag and a C-terminal His tag (GST-VP4, Figure 1A) was generated and its bacterial expression was analyzed.

The GST-VP4 construct was effectively expressed in *E. coli* after induction, however, the protein accumulated in the insoluble protein fraction indicative of its appearance in intracellular inclusion bodies (data not shown). Bacteria sequester insoluble proteins into intracellular inclusion bodies to maintain cellular homeostasis [11]. Since proteins found in inclusion bodies are commonly misfolded and therefore likely inactive, strategies for optimizing the production of soluble GST-VP4 were explored.

Osmolytes such as proline can be used to stabilize proteins in cells [12], [13]. The addition of proline (20 mM) and high concentrations of salt (NaCl 300 mM) to the medium followed by growth at the reduced temperature of 30°C significantly increased the solubility of GST-VP4 (Figure 1B, lanes 3 and 4). High concentrations of salt in the culture media induce *E. coli* to concentrate proline *in vivo* and decrease the tendency of some proteins to aggregate [14]. GST-VP4 from the soluble fraction was purified to homogeneity utilizing sequential interactions with its dual affinity tags (Figure 1C, lane 6). Size-exclusion chromatography demonstrated that GST-VP4 was monomeric (Figure S1). The cleavage of GST from VP4 with TEV protease produced the two corresponding proteins GST and VP4 (Figure 1C, lane 7). However, the release of GST from VP4 led to the aggregation of VP4. Therefore, purified uncleaved GST-VP4 was employed for all subsequent studies.

### VP4 possesses hemolytic activity

**Figure 2:**
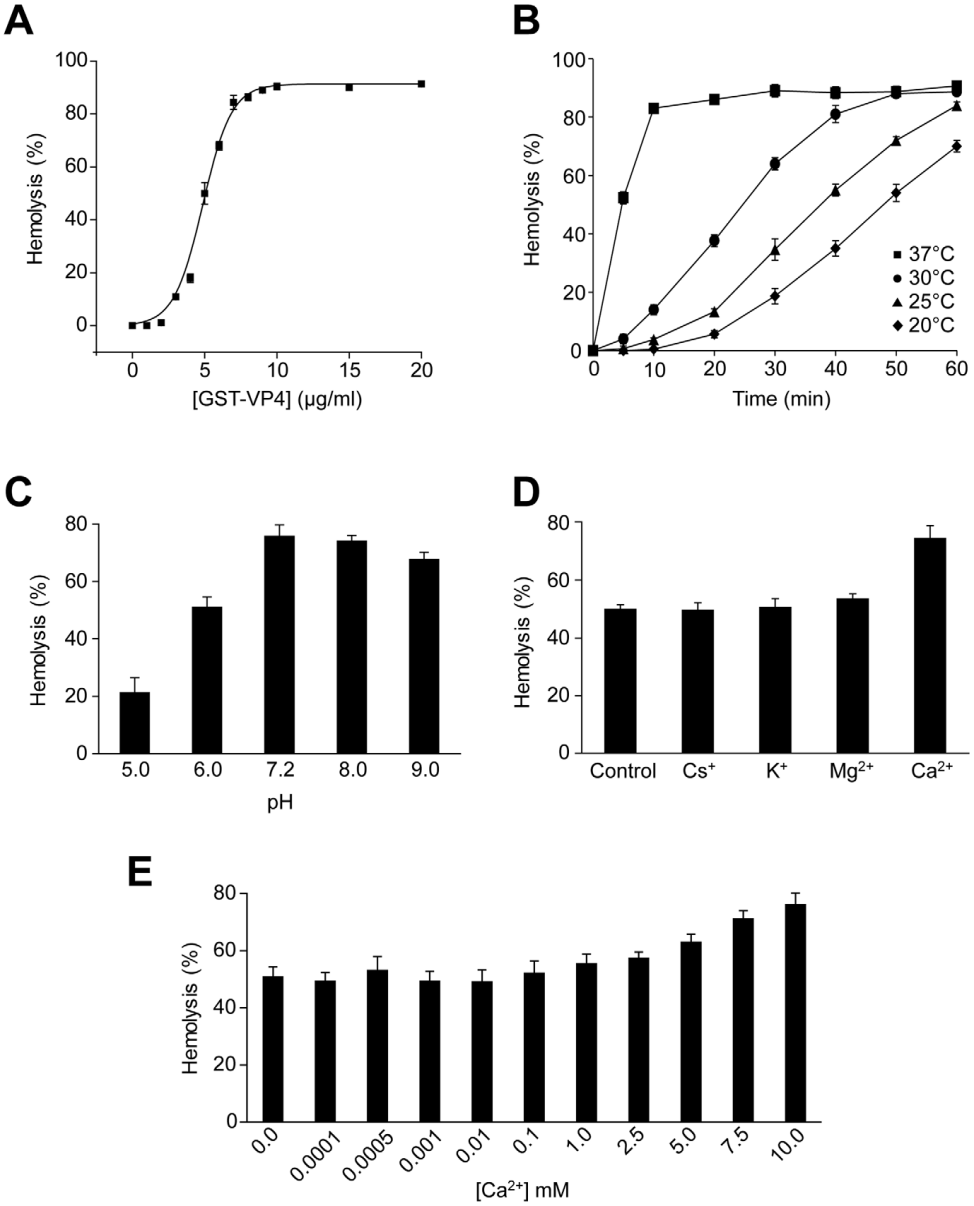
VP4 possesses hemolytic activity. (A) Percent hemolysis of bovine RBCs as a function of GST-VP4 concentration. RBCs (0.5%, v/v) were incubated with various concentrations of the protein for 30 min at 37°C. (B) Kinetics of hemolysis of bovine RBCs as a function of temperature as indicated. RBCs were incubated at different temperatures with GST-VP4 (10 µg/ml) for 30 min. (C) Hemolysis as a function of pH. Hemolysis reactions were carried out at varying pH. Data are normalized with respect to samples containing only buffer and RBCs (0%), and containing RBCs with 50% water to mediate complete lysis (100%). (D) Hemolysis in presence of different metal ions. Purified GST-VP4 was mixed with bovine RBCs. Reactions were incubated in 10 mM Tris (pH 7.5)/150 mM NaCl containing Cs^+^, K^+^, Mg^2+^, Ca^2+^ ions. (E) Calcium concentrations were varied for the hemolytic assay over a wider range. The error bars represent the standard deviation from three independent experiments.

To determine whether VP4 displays membrane disruptive activity, a hemolysis assay was employed using bovine RBCs. RBCs constitute a simple and efficient model system to study protein-membrane interactions and lysis. RBCs were treated with various concentrations of purified GST-VP4 at 37°C for 30 min. The level of hemolysis was measured by determining the fraction of hemoglobin released into the supernatant after centrifugation. GST-VP4 mediated hemolysis was found to be concentration dependent. GST-VP4 efficiently lysed bovine RBCs in a concentration dependent manner inducing 50% lysis at a concentration of 5 µg/ml (Figure 2A). The equilibrium value of percent hemolysis showed sigmoidal dependence on GST-VP4 concentration, which is a characteristic feature of a process dependent on self-oligomerization [15]. Maximum hemolysis was observed with concentrations of 10 µg/ml of GST-VP4 or higher.

To determine the temperature dependency of hemolysis, time courses for hemolysis were studied at different temperatures using 10 µg/ml of GST-VP4. Aliquots were removed at the indicated times and percent hemolysis was determined. GST-VP4 rapidly and efficiently lysed the RBCs at 37°C (Figure 2B). The maximal level of hemoglobin was released after 10 min of treatment at 37°C. At the lower temperatures, hemolysis occurred after a lag phase of 5 to 15 min. This lag phase period increased as the temperature was reduced. In addition, the maximal level of hemolysis diminished as the temperature was lowered. This suggests that the disruption of RBCs by GST-VP4 involved a temperature dependent rate-limiting step.

The presence of metal ions and pH has been reported to influence the hemolytic activity of viroporins and bacterial pore-forming toxins [16], [17]. To further analyze the hemolytic activity of VP4, the metal ionic and pH dependency of the GST-VP4 mediated hemolytic reaction was characterized. Hemolysis was unaffected by the presence of Cs^+^, K^+^ or Mg^2+^ ions (Figure 2D). However, hemolytic activity increased by 25% in the presence of Ca^2+^. As biological levels of Ca^2+^ are regulated and vary over a large range from nM to mM [18], a broader range of calcium concentrations were tested for their effect on VP4 mediated hemolysis. Moderate increases in hemolysis were observed in the presence of 5 mM or higher concentrations of Ca^2+^ (Figure 2E). In addition, maximal hemolytic activity for GST-VP4 was observed at pH 7.2 (Figure 2C). The hemolytic activity of GST-VP4 was dramatically reduced in acidic conditions and mildly decreased in alkaline conditions. Altogether, GST-VP4 showed optimal membrane disruption activity at 37°C in the presence of mM calcium levels at neutral pH. All studies that follow were performed at the optimal temperature of 37°C and neutral pH.

### Deletion of hydrophobic domain of VP4 impairs its ability to bind and disrupt cellular membranes

**Figure 3:**
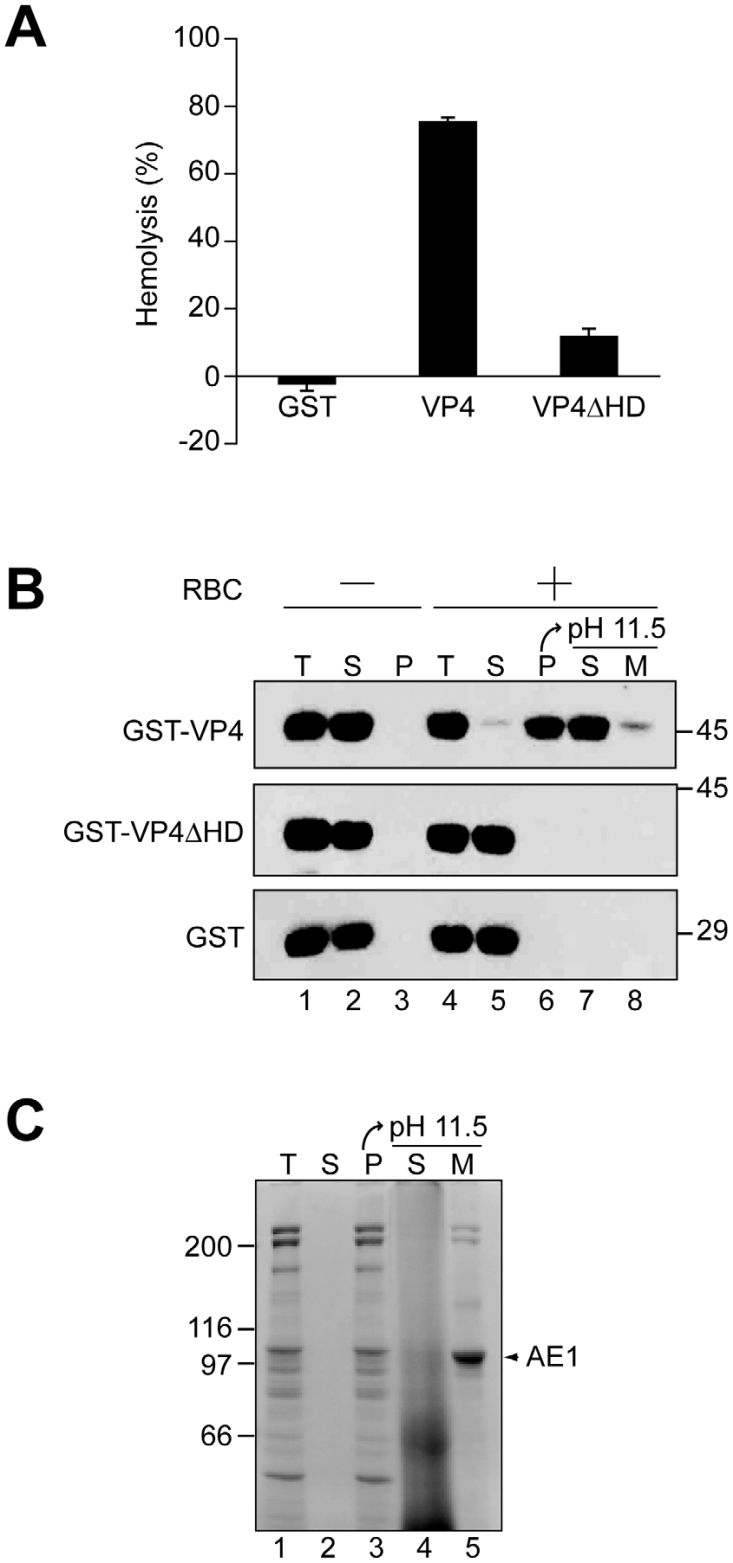
The hydrophobic domain of VP4 is required for its binding and disruption of RBC membranes. (A) GST-VP4 or GST-VP4ΔHD were incubated with bovine RBCs for 30 min at 37°C. Released hemoglobin was measured by the A_414_ of the supernatant after centrifugation and the removal of unlysed cells. GST was used as a control to rule out its contribution in the hemolytic activity of GST-VP4. (B) Hemolysis reaction mixtures (lane 4, T) containing bovine RBCs and GST-VP4 or GST-VP4ΔHD were incubated at 37°C for 30 min. RBC bound (P, lane 6) and unbound (S, lane 5) proteins were separated by centrifugation. Membrane fractions (lane 6) were alkaline extracted with 0.1 M Na_2_CO_3_, pH 11.5 and ultracentrifuged to separate the soluble (S) and membrane (M) fractions (lane 7 and 8). Samples resolved by reducing SDS-PAGE were immunoblotted with antibody against GST. Separate reactions were performed in the absence of RBCs (lanes 1–3). (C) Bovine RBCs were separated as in B and the SDS-PAGE gel was stained with coomassie blue to visualize the abundant RBC membrane protein, anion exchanger 1 (AE1).

Hydrophobic stretches of ∼20 amino acids in length can interact with membranes to form transmembrane segments [19]. To establish the role of the hydrophobic domain of VP4 in its ability to perturb membranes, the hydrophobic domain was deleted from GST-VP4 (GST-VP4ΔHD) and the membrane disruptive activity of the deletion mutant was analyzed. GST-VP4ΔHD was expressed and purified in the same way as GST-VP4 (Figure 1B and C). Deletion of the hydrophobic domain significantly reduced the membrane disruptive activity of VP4, indicative of the hydrophobic domain playing a critical role in plasma membrane lysis (Figure 3A). GST alone did not display membrane disruptive activity, verifying that the late viral protein VP4 possessed hemolytic or membrane disruption activity (Figure 3A). Furthermore, hemolytic activity of VP4 was also abolished when the hydrophobic domain was substituted with either of two well-characterized transmembrane segments from bacterial leader peptidase [20], [21], demonstrating that the hydrophobic domain of VP4 is specifically required for its lytic activity (Figure S2A and Text S1).

A cell-binding assay was employed to determine if VP4 interacts with cell membranes. GST-VP4, GST- VP4ΔHD and GST were separately incubated without and with RBCs for 30 min at 37°C, and bound and unbound fractions were separated by centrifugation. Cell binding was determined by the amount of protein that sedimented with the cells. In the absence of RBCs, all of the proteins tested remained soluble and were therefore found in the supernatant (Figure 3B, lanes 2 and 3). However, in the presence of RBCs, only GST-VP4 was localized to the RBC pellet, indicative of the efficient binding of VP4 to RBCs (Figure 3B, lane 6). Both GST- VP4ΔHD and GST remained in the supernatant after centrifugation demonstrating the necessity for the hydrophobic domain of VP4 for cell binding.

To determine if the bound protein was integrated into the lipid bilayer of the cells, the bound fractions were alkaline extracted with membrane and soluble fractions separated by ultracentrifugation [22]. After alkaline extraction, the abundant RBC membrane protein, anion exchanger 1 (AE1), was found in the membrane pellet (Figure 3C, lane 5) [23]. Interestingly, the vast majority of the membrane associated GST-VP4 (88%) was found in the supernatant after alkaline extraction and centrifugation (Figure 3B, lane 7 compared to 8). This suggested that GST-VP4 was not fully integrated into the lipid bilayer.

### RBC proteins are not required for VP4-mediated hemolysis

**Figure 4:**
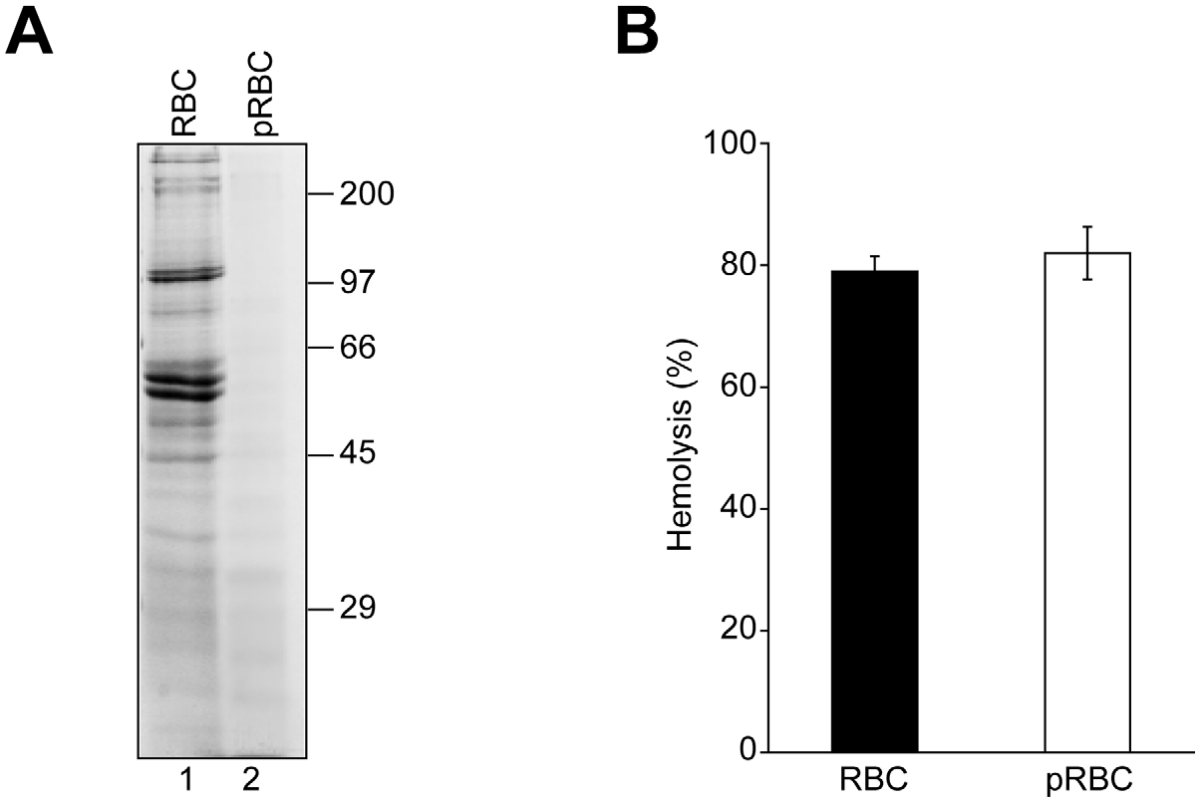
RBC surface proteins are not required for VP4-mediated hemolysis. (A) RBCs (lane 1) were pretreated with trypsin and proteinase K (pRBCs, lane 2) to remove surface proteins. Membrane fractions after hypotonic lysis were resolved by reducing SDS-PAGE and proteins were visualized by coomassie blue staining. (B) GST-VP4 was mixed with untreated or protease treated RBCs. After incubation for 30 min at 37°C, hemoglobin release was measured by the A_414_ of the supernatant after centrifugation to remove unlysed cells. The error bars represent the standard deviation from two independent experiments performed in triplicate.

We next investigated if GST-VP4 required RBC membrane proteins for its hemolytic activity. To remove extracellular-exposed RBC proteins that could potentially serve as a platform for VP4 binding, RBCs were treated with both proteinase K and trypsin to generate protease-treated RBCs (pRBC). Membrane fractions from treated and untreated RBCs after hypotonic lysis to remove intracellular proteins were isolated by centrifugation. The protein content of the membrane fractions was monitored by SDS-PAGE. Numerous proteins corresponding to a wide range of sizes were observed in the untreated sample, but proteins were absent from the protease treated sample demonstrating the effectiveness of the protease treatment (Figure 4A). Protease treatment of RBCs or the removal of the surface proteins did not affect the GST-VP4 mediated hemolysis reaction (Figure 4B). Surface proteins were not required for the membrane disruptive activity of GST-VP4. This suggested that GST-VP4 interacted with the plasma membranes of the RBCs directly via the lipids.

### VP4 permeabilized liposomal membranes

**Figure 5:**
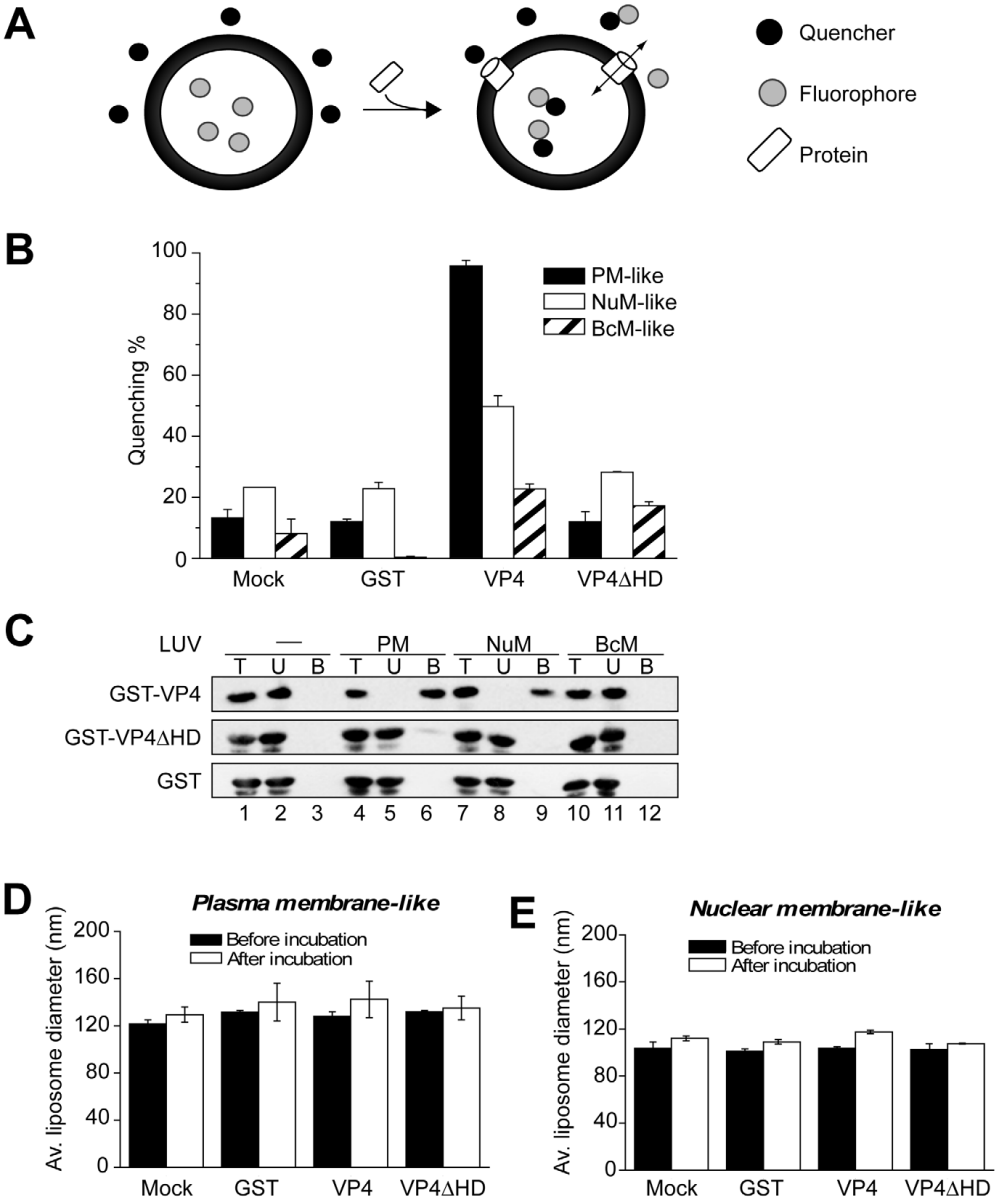
VP4 permeabilization of liposomes. (A) Scheme showing the liposome disruption assay employed. Membrane disruption was examined by encapsulating [Tb(DPA)^3-^_3_] fluorophore into LUVs (large unilamellar vesicles). When these LUVs were resuspended in a solution containing EDTA (quencher), protein mediated membrane disruption was monitored by the quenching of [Tb(DPA)^3-^_3_] emission as the encapsulated molecules were released, and terbium ions were chelated by EDTA. (B) VP4 disrupts mammalian plasma (PM-like) and nuclear membrane-like (NuM-like) LUVs. LUVs mimicking the lipid compositions of bacterial inner membrane (BcM-like), and mammalian plasma and nuclear membranes were prepared to examine the membrane disruption activity of GST-VP4 and GST-VP4ΔHD. GST was used as a control. Mock LUVs were incubated in absence of any protein. Liposome disruption was evaluated using LUVs prepared with selected lipid compositions, and the percentage of fluorophore quenched is indicated. Each data point shows the average of at least two independent measurements with error bars representing standard deviations. (C) Flotation of proteins on sucrose gradients after incubation either without (-, lanes 1–3) or with PM-like (lanes 4–6), NuM-like (lanes 7–9), or BcM-like (lanes 10–12) LUVs to separate unbound (U; lanes 2, 5, 8 and 11) and bound (B; lanes 3, 6, 9 and 12) fractions. Proteins were resolved by SDS-PAGE and immunoblotting with anti-GST antibody. (D) Average diameter of PM-like LUVs before and after 30 min incubation with the indicated proteins as determined by dynamic light scattering. Each data point shows the average of at least two independent measurements with error bars representing standard deviation. (E) Average diameter of NuM-like LUVs determined similarly to D.

To verify that VP4 acts on lipids directly, a membrane disruption assay was employed that utilized liposomes with lipid compositions representative of various biological membrane sources. This fluorescence-based spectroscopic assay detected the release of liposome-encapsulated fluorophores following the addition of GST-VP4. Bathing the liposomes in quenchers of the encapsulated fluorophore supports a reduction of fluorescence intensity if the fluorophore is released from the liposomes or the quencher is allowed to enter the liposome as a result of membrane disruption permitting contact between the quencher and the fluorophore (Figure 5A). In contrast, quenching is not observed if the membrane remains intact and the quencher and the fluorophore are unable to cross the membrane bilayer [24]. This experimental system provides a highly tractable approach to characterize the membrane disruption properties of VP4.

Large unilamellar vesicles (LUV) mimicking the lipid compositions of bacterial, and mammalian plasma and nuclear membranes were prepared to examine the membrane disruption activity of GST-VP4 (Table 1) [25], [26], [27]. GST-VP4 efficiently disrupted mammalian plasma (PM-like) and nuclear membrane-like (NuM-like) liposomes (Figure 5B). The activity against PM-like liposomes was approximately double that which was directed against NuM-like liposomes. The viral protein did not affect liposomes mimicking the lipid composition of the bacterial inner membrane (BcM-like). Furthermore, regardless of the liposomes tested, the hydrophobic domain was found to be essential for the membrane disruption activity of VP4, as VP4ΔHD and GST alone displayed no significant membrane disruption. These results demonstrated that VP4 possessed membrane-permeabilizing activity that required its hydrophobic domain and its activity was optimal against membranes that represented the lipid composition of the mammalian plasma membrane.

**Table 1:** Lipid composition of liposomes.

Liposomes	Chol	PC	PE	SM	PS	PG
Bacterial (*E. coli*) inner membrane (BcM-like)	0	0	80	0	0	20
Mammalian plasma membrane (PM-like)	50	20	11	13	6	0
Mammalian nuclear membrane (NuM-like)	15	51	20	9	5	0
NuM-like-noPE	19	64	0	11	6	0
NuM-like+Chol	50	31	12	5	3	0
PC	0	100	0	0	0	0
Chol+PC	40	60	0	0	0	0
Chol+PC+PE	40	40	20	0	0	0
Chol+PC+SM	40	45	0	15	0	0
Chol+PC+PE+SM	40	25	20	15	0	0

The lipid compositional data are expressed as a molar percentage of the total lipid. PC, phosphatidylcholine; PE, phosphatidylethanolamine; SM, sphingomyelin; PS, phosphatidylserine; PG, phosphatidylglycerol; Chol, cholesterol.

To explore if the differences observed for liposome disruption were caused by the ability of VP4 to bind to the various membranes, a membrane-binding assay was employed. A liposome flotation assay was used to isolate liposomes and monitor VP4 binding. Bound and unbound fractions of VP4 were quantified by immunoblotting. VP4 bound efficiently to both PM-like and NuM-like liposomes and did not display any association with BcM-like liposomes (Figure 5C, lanes 6, 9 and 12). These results were consistent with the fluorescence-based liposome disruption assay. In addition, VP4ΔHD mutant and GST did not bind any of the liposomal membranes (Figure 5C). Therefore, the liposome binding results indicate that the membrane disruption activity required stable membrane binding.

To investigate the origin for the difference in the activities of VP4 to the various membranes, liposomes comprised of different lipid compositions were tested to determine which lipids affected the membrane disruption properties of VP4. VP4 was most active against PM-like liposomes, followed by NuM-like liposomes, while it displayed marginal activity against BcM-like liposomes. Since BcM-like liposomes were rich in phosphatidylethanolamine (PE) and contained no cholesterol, the influence of PE and cholesterol on the activity of VP4 was tested. Given that VP4 supported 50% fluorescence quenching in NuM-like liposomes, the composition of these liposomes was modified in an attempt to optimize membrane disruption by VP4.

**Figure 6:**
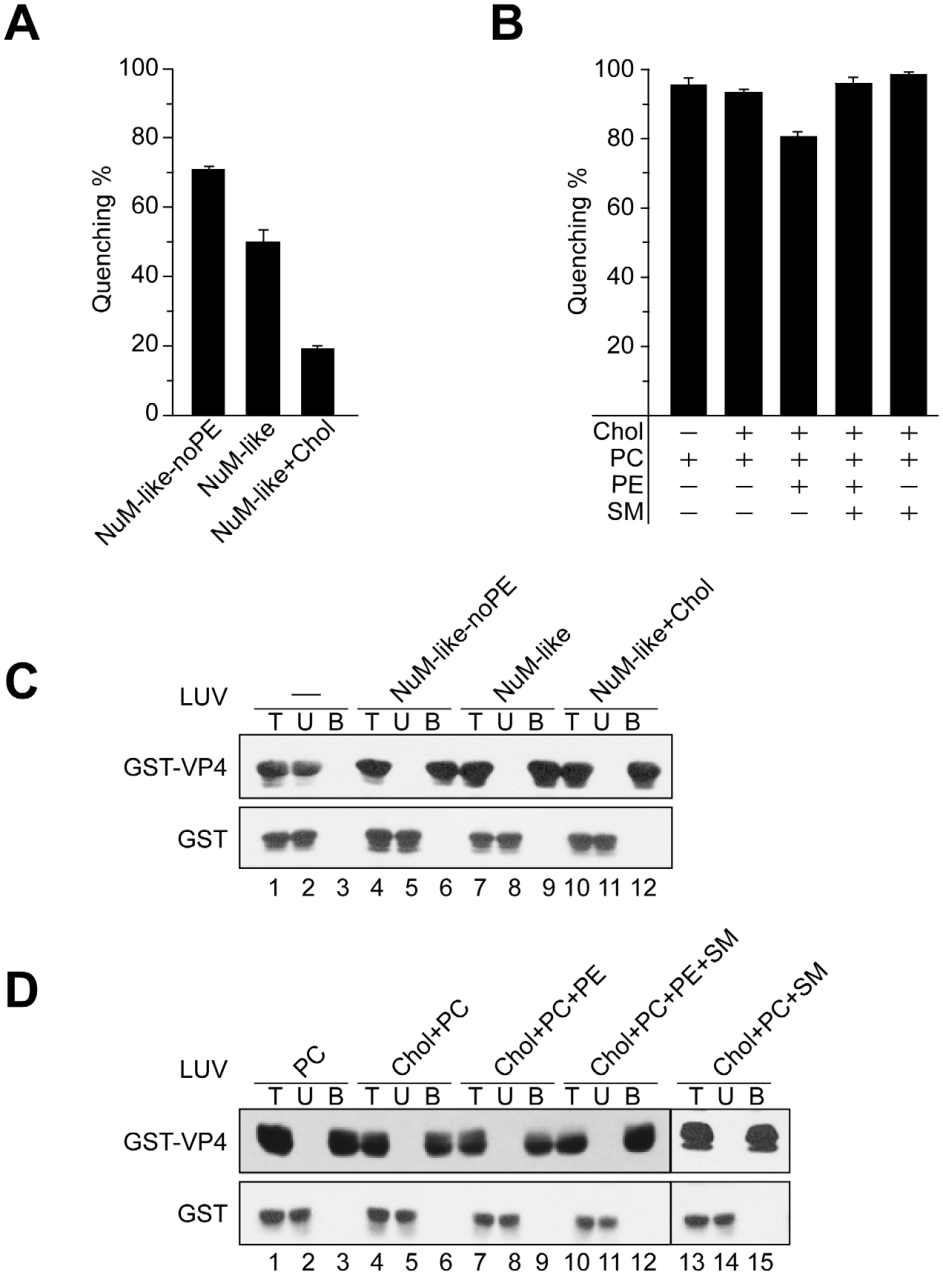
Activity of VP4 is dependent upon the lipid composition of the membranes. (A) Membrane disruptive activity of GST-VP4 measured as percentage fluorescence quenching with two sets of LUVs where phosphatidylethanolamine (PE) was excluded from NuM-like LUVs (NuM-Iike-noPE) or the percentage of cholesterol was increased (NuM-Iike+Chol). Each data point shows the average of at least two independent measurements and the error bars denote the standard deviation of the experiment. See Table 1 for lipid fractions. (B) Membrane disruptive activity of GST-VP4 with five different sets of LUVs: PC, Chol+PC, Chol+PC+PE, Chol+PC+PE+SM and Chol+PC+SM. Each data point shows the average of at least two independent measurements and the error bars denote the standard deviation. (C) Flotation of proteins on sucrose gradients after incubation either without (-, lanes 1–3) or with NuM-like-noPE (lanes 4–6), NuM-like (lanes 7–9), or NuM-like+Chol (lanes 10–12) LUVs to separate unbound (U; lanes 2, 5, 8 and 11) and bound (B; lanes 3, 6, 9 and 12) fractions. Proteins were resolved by SDS-PAGE and immunoblotting with anti-GST antibody. (D) Flotation of proteins on sucrose gradients after incubation with PC (lanes 1–3), Chol+PC (lanes 4–6), Chol+PC+PE (lanes 7–9), Chol+PC+PE+SM (lanes 10–12), or Chol+PC+SM (lanes 13–15) LUVs. Proteins were resolved by SDS-PAGE and immunoblotting with anti-GST antibodies.

VP4-mediated membrane disruption increased when PE was excluded from the NuM-like liposomes (Figure 6A, NuM-like-noPE). This suggested that PE might inhibit membrane perturbation by VP4. The addition of cholesterol to the NuM-like liposomes (NuM-like+Chol) caused a significant decrease in membrane permeabilization. Cholesterol did not appear to produce a direct inhibitory effect as no difference was observed between the quenching of phosphatidylcholine (PC) liposomes and cholesterol+PC-containing liposomes (Figure 6B, Chol+PC). VP4 showed highest activity against PM-like membranes that contained 50% cholesterol, again suggesting that cholesterol did not have a direct inhibitory effect on VP4 activity (Figure 5B).

Increasing cholesterol levels in NuM-like liposomes was also associated with a decrease in the levels of PC and sphingomyelin (SM) (Table 1). Therefore, the level of SM and PE was varied while keeping the cholesterol content constant. First, the addition of PE to the cholesterol+PC liposomes produced a decrease in quenching (Figure 6B), consistent with the previously discussed inhibitory effect of PE. Interestingly, this inhibitory effect was reversed by the addition of SM (Figure 6B). As VP4 efficiently bound to all liposomes tested besides the bacterial-like liposomes that were rich in PE (Figure 5C, 6C and 6D), high concentrations of PE appeared to inhibit VP4 binding. Altogether these results showed that VP4 permeabilizing activity was dependent on the membrane lipid composition and VP4 was most active against PM-like liposomes due to what appeared to be a combined effect of lower PE and higher SM levels.

### VP4 forms small size selective pores in biological membranes

The observed release of liposomal content by VP4 led to the question of whether VP4 lysed the liposomes by creating discrete pores in the membrane or by a non-specific membrane solubilization or a detergent-like effect. In support of the pore formation hypothesis, LUVs average diameter or their total scatter intensity did not change significantly after incubation with VP4 (Figure 5D, 5E and data not shown). This indicated that the liposomes were not solubilized by the viral protein.

To further explore the characteristics of the pores formed by VP4, the pore forming activity of VP4 on cellular membranes was tested by analyzing the osmoprotection capabilities of different size polyethylene glycols (PEGs). The rationale for this approach is that pores at the plasma membrane produce cell lysis by uncontrolled water and ion influx. PEGs can serve as osmotic protectants and prevent water and ion influx, only if the PEG molecules are larger than the size of the VP4 formed pore. In contrast, if PEGs are small enough to pass through pores formed by VP4, their concentration equilibrates rapidly across the membrane and no osmotic protection is conferred, resulting in cell lysis. Therefore, the presence of discrete-sized pores in the membrane and their approximate diameter can be estimated by determining the minimum size PEG that confers osmoprotection [28].

**Figure 7:**
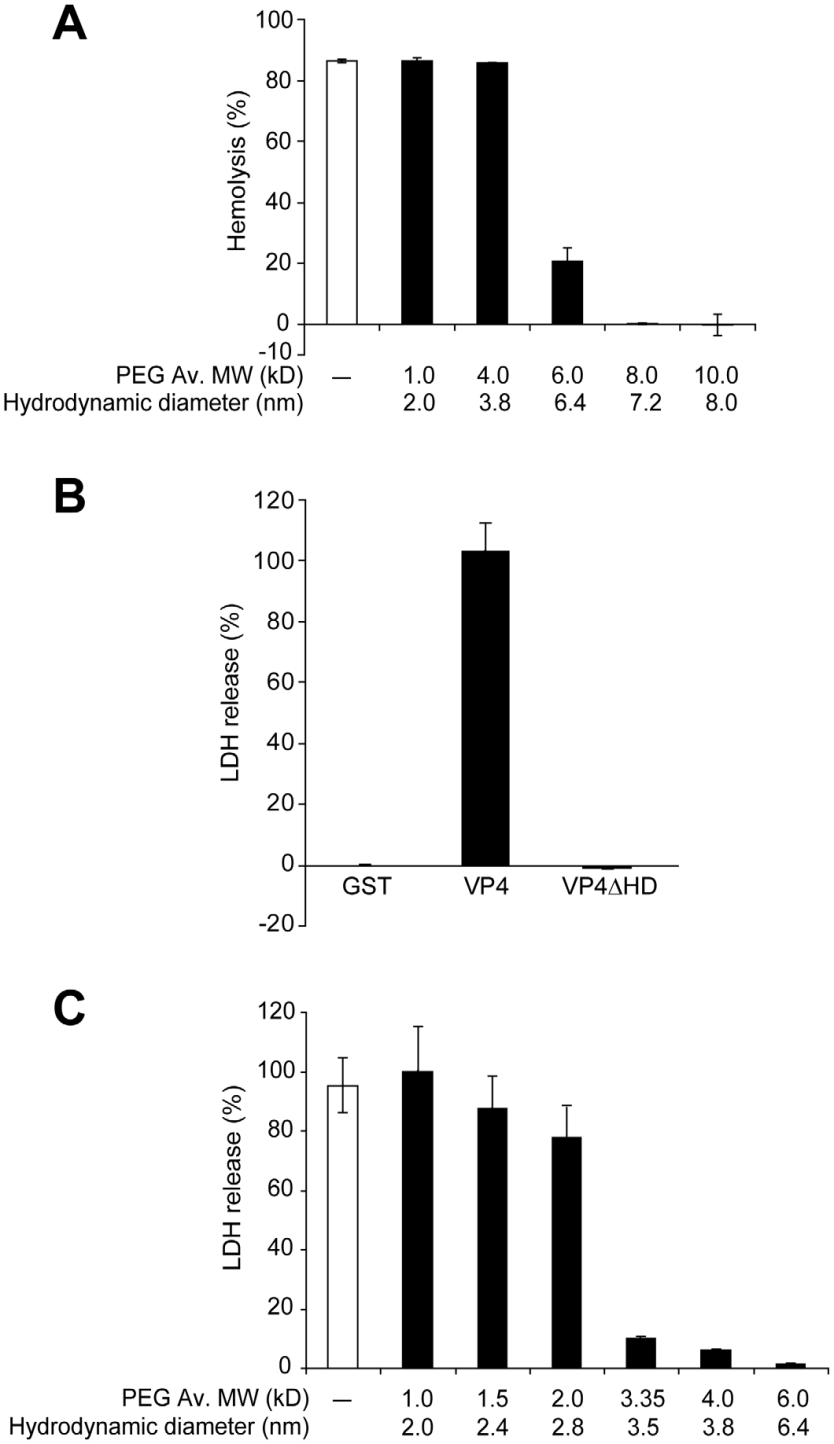
VP4 forms small size selective pores in biological membranes. (A) Assessment of osmoprotection of red blood cells from lysis by VP4. Bovine RBCs (0.5%, v/v) were mixed with GST-VP4 (10 µg/ml) in the presence of polyethylene glycols (PEGs) of increasing molecular weights (1.0, 4.0, 6.0, 8.0 and 10.0 kD). The error bars represent the standard deviation from three independent experiments. (B) Percentage LDH released from Cos 7 cells after incubation with VP4 and VP4ΔHD for 30 min at 37°C. The percentage of LDH released was calculated by dividing the LDH released from samples by the LDH released with Triton X-100-permeabilized Cos 7 cells incubated under the same conditions (see Materials and Methods). The error bars represent the standard deviation from three independent experiments each carried out in triplicate. (C) Assessment of osmoprotection of Cos 7 cells from lysis by GST-VP4. Cos 7 cells were mixed with GST-VP4 (10 µg/ml) in the presence of different molecular weights PEGs (1.0, 1.5, 2.0, 3.35, 4.0 and 6.0 kD). The error bars represent the standard deviation from three independent experiments performed in triplicate.

The hemolytic activity of GST-VP4 was examined in the presence and absence of 30 mM PEGs. Smaller PEGs (1 and 4 kD) had negligible osmoprotection effect on hemolysis, whereas PEGs of 6 kD and larger reduced hemolysis (Figure 7A). These results supported the hypothesis that VP4 formed pores in RBC membranes. A sharp transition in osmoprotection was observed between PEGs of 4 and 6 kD with estimated hydrodynamic diameters of 3.8 nm and 6.4 nm, respectively. This suggested that VP4 formed ∼4–6 nm diameter pores in the plasma membrane of bovine RBCs.

The pore formation properties of VP4 were examined using Cos 7 cells, a SV40 permissive host cell line. Cell lysis can be followed by the release of cytosolic proteins. After VP4 treatment, the release of cytoplasmic lactate dehydrogenase (LDH) from Cos 7 cells was used to probe cell lysis. Incubation of Cos 7 cells at 37°C for 30 min led to the complete release of intracellular LDH (Figure 7B). Consistent with hemolysis and liposome disruption results, the deletion of the hydrophobic domain of VP4 abolished its cytolytic activity.

The cytolytic activity of VP4 on Cos 7 cells was analyzed using different size PEGs. PEGs larger than 3.35 kD efficiently inhibited cytolysis (Figure 7C). There was a gradual reduction in VP4-mediated cytolysis in the presence of PEG from 1 to 2 kD in size. These results suggested that VP4 formed smaller pores in the plasma membrane of Cos 7 cells. The transition in osmoprotection occurred with PEGs between 2 and 3.35 kD in size, which corresponded to hydrodynamic diameters of 2.8 and 3.5 nm, respectively. This indicated that VP4 formed pores of ∼3 nm in diameter in the cell membrane of the SV40 permissive host cells.

## Discussion

VP4 is a late expressed SV40 hydrophobic protein proposed to play a role in viral release [9]. Here, the membrane disruption properties of purified VP4 were thoroughly characterized. VP4 efficiently bound and permeabilized bovine RBCs. The hydrophobic domain of VP4 was required for these activities, which were optimal at 37°C and neutral pH in the presence of calcium. The disruption of LUV by VP4 demonstrated that VP4 had a preference for liposomes comprised of lipid compositions representing plasma and nuclear membranes. The membrane disruption activity of VP4 supported the formation of ∼3 nm pores in the plasma membranes of RBCs and the permissive SV40 host (Cos 7 cells). These results are consistent with VP4 causing the lysis of infected cells to support the poorly understood process of nonenveloped viral release.

VP4 disrupted membranes by directly interacting with the membrane lipid bilayer and its membrane disruption ability was dependent upon the composition of the bilayer. VP4 efficiently lysed bovine RBCs, which are rich in SM [29]. In liposome (or LUV) studies, SM rescued VP4 activity in the presence of the inhibitory lipid PE. Bacterial membranes contain high levels of PE. The inability of GST-VP4 to lyse PE-rich or BcM-like liposomes likely fortuitously contributed to the expression of large amounts of the protein in bacteria. PE is a conical lipid that induces negative membrane curvature, which might contribute to its inability to support VP4 pore formation [30], [31]. Pore formation by the antimicrobial peptide from the *Xenopus* skin, magainin 2, was induced by positive membrane curvature and inhibited by negative membrane curvature [30]. The membrane disruption activity of VP4 did not appear to be influenced by cholesterol, whereas high levels of PC favored efficient pore formation. Both nuclear and plasma membranes are rich in PC. While GST-VP4 disrupted liposomes that were representative of both plasma and nuclear membranes, it displayed a strong preference for PM-like liposomes. This was consistent with its ability to efficiently lyse RBCs and Cos 7 cells. The membrane disruption activity of VP4 was highly influenced by the lipid composition of the LUV.

Enveloped and nonenveloped viruses utilize proteins termed viroporins (viral-encoded membrane pores) to mediate membrane disruption events during the viral life cycle. Currently over a dozen viroporins are known and their functions appear to be similar to the well-studied toxins from bacterial pathogens such as *Bacillus anthracis* protective antigen (PA63) and *E. coli* (hemolysin) that form membrane pores [32]. Typically, viroporins are small (60–120 residues), hydrophobic proteins that form oligomeric structures in lipid bilayers of infected cells. These viral-encoded proteins form hydrophilic pores in host cell membranes to modify their permeability or stability. The membrane pores support the movement of ions or small molecules across membrane bilayers, to potentially aid in the viral entry and penetration steps, or promote the efficient release of virions by compromising the integrity of host cell membranes [33].

We demonstrated that VP4 acts as a viroporin. *In vitro* translated VP4 efficiently bound to GST-VP4 suggestive of the ability of VP4 to oligomerize (Figure S2B), a property shared by viroporins. Viroporins also frequently contain stretches of basic amino acids that are proposed to act as detergents by potentially binding to anionic lipid head groups [33]. VP4 possesses a basic pI of 10.2 and a large number of basic residues disproportionately clustered to the C-terminal side of the hydrophobic domain. These basic residues include a nuclear localization sequence, shared by both VP2 and VP3. While the lipid composition and the hemolytic activity of VP4 are consistent with its ability to associate with and disrupt plasma membranes, it will be of interest to determine what cellular membranes VP4 interacts with when it is expressed in the cytoplasm of host cells, as is the case during viral infection. Previous results found that VP4 appeared to accumulate at the nuclear periphery upon transfection of the SV40 viral genome lacking VP2 and VP3 [9]. The directionality of the VP4 membrane binding and disruption in the hemolysis reaction differs from that utilized during the viral life cycle. The plasma membrane is asymmetric, possessing higher concentrations of phosphatidylserine (PS) on the inner facing membrane leaflet. Since PS did not affect VP4 pore forming activity, this membrane asymmetry was not likely to affect the membrane permeabilization assay. These results indicated that VP4 effectively disrupted membranes that mimicked plasma and nuclear membrane compositions, with a preference for plasma-like membranes.

The protein-mediated mechanisms for membrane lysis have been studied extensively using antimicrobial peptides, which are lytic substances secreted by cells for a means of defense against microbial pathogens [34]. Antimicrobial peptides can contain short cationic and hydrophobic sequences that lack Cys residues, like VP4. Antimicrobial peptides have been proposed to disrupt bacterial membranes using three possible mechanisms. First, the barrel-stave model involves the formation of transmembrane pores created by alpha-helices integrated into the bilayer. This mechanism of membrane perturbation is unlikely for VP4 as VP4 was extracted from membranes after alkaline treatment, indicating that it was not fully integrated into the bilayer (Figure 3B). Secondly, in the carpet model, peptides accumulate on the membrane surface through electrostatic forces (cationic proteins binding anionic lipid head groups). At high concentrations, it is proposed that these peptides disrupt the membrane in a detergent-like manner resulting in the formation of micelles. The sharp size distribution in the pores formed by VP4 (Figure 7A and C), and the invariable diameter of the LUVs observed by dynamic light scattering (Figure 5D and E) after VP4 treatment are not consistent with VP4 acting through a carpet model since complete membrane disruption or lysis was not observed. Finally for the toroidal-pore model, proteins insert into the membrane and by interacting with the lipid head groups force curvatures in the interacting lipids, resulting in the fusion of the inner and outer leaflets at the lipid-protein interaction site. The toroidal-pore model differs from the barrel-stave model in that the protein interacts mostly with the lipid head groups and is not directly inserted through the hydrophobic core of the membrane [35]. The sharp pore size distribution and the ability to extract VP4 from membranes after alkaline treatment are consistent with VP4 forming a toroidal-pore within the membrane. However, further studies will be needed to fully delineate the membrane disruption mechanism of VP4.

Cytolytic viruses such as the nonenveloped polyomaviruses and picornaviruses release their viral progeny by initiating the timely lysis of host cells. The release of viral particles by cell lysis after adequate numbers of viral particles have been assembled ensures the efficient spread of the virus. Previously, we identified SV40 VP4 as a protein encoded within the viral genome that is expressed in the host cell at later times during infection that coincide with viral release [9]. The SV40 virus has a diameter of 50 nm therefore it is too large to be directly translocated through the ∼3 nm VP4 pores formed in Cos 7 cells. Whereas GST-VP4 efficiently forms pores in mammalian cells, at this time we cannot rule out the possibility that the size of the pore is influenced by the GST attached to its N-terminus. However, we favor the explanation that VP4 pores alter the cytoplasmic concentration of ions or other small molecules, which leads to cell lysis. This appears to be the mechanism for the 2B protein mediated release of picornavirus [36]. Alternatively, VP4 may form heterocomplexes involving other viral proteins that influence the size of the pores formed. Recently for JC virus (a human polyomavirus), the viral encoded agnoprotein was identified as a viroporin that aids in the release of JC virus [37]. As the SV40 genome also encodes for the hydrophobic agnoprotein, it is possible that these proteins work in concert to initiate viral release.

VP4 is an N-terminal truncation of the late structural viral proteins VP2 and VP3 [9]. Our previous studies found that VP3 and VP4 co-expression supported bacterial lysis suggesting that heterocomplexes between other late proteins and VP4 may influence lipid specificity or the size of the pores formed. While VP4 is solely found in infected cells [9], VP2 and VP3 are minor structural components of the viral particle [5], [6]. Upon internalization, SV40 traffics to the endoplasmic reticulum, the proposed site of uncoating and penetration [38]–[41]. An important issue for the penetration of nonenveloped viruses is how does a subviral particle or the viral genome cross endomembranes without disrupting cellular homeostasis so that the cell can be exploited for viral production for subsequent hours or days [42], [43]. VP2 and VP3 have been shown to insert into ER membranes [22]. This leads to the provocative possibility that since VP2 and VP3 both possess the VP4 sequence involved in membrane disruption, the exposure of VP2 and VP3 after viral uncoating supports membrane disruption to permit viral penetration. Interestingly, VP2 and VP3 from SV40 and polyomavirus have been shown to exhibit membrane disruption activities [44], [45]. Future studies will be required to address the properties of VP2/VP3 and VP4-heterocomplexes, and their roles in viral penetration and release.

## Materials and Methods

### Reagents

The GST-Tag (12G8) mouse monoclonal antibody was purchased from Abmart (Arlington, MA). CytoTox 96 cytotoxicity assay kit for LDH release determination was purchased from Promega (Madison, WI). All phospholipids were obtained from Avanti Polar Lipids (Alabaster, AL), while cholesterol was obtained from Steraloids (Newport, RI). AcTEV protease was purchased from Invitrogen (Carlsbad, CA). All other reagents were purchased from Sigma (St. Louis, MO).

### DNA constructs

The pGEX-6P-1 plasmid (Amersham Bioscience; Piscataway, NJ) was modified to include a tobacco etch virus (TEV) protease site and a C-terminal 6xHis epitope upstream and downstream of the multiple cloning site, respectively, to create pGEX-6P-1-TEV-His. The GST-tagged version of full length VP4 was created by PCR cloning into the bacterial expression plasmid pGEX-6P-1-TEV-His using standard techniques. VP4 contained an N-terminal GST tag and a C-terminal His tag (GST-TEV-VP4-His). The QuikChange mutagenesis primer design program was used to delete the hydrophobic domain of VP4 (amino acids 65–83, PQWMLPLLLGLYGSVTSAL) to create VP4ΔHD. Mutagenesis was confirmed by sequencing.

### Recombinant protein expression and purification

The BL21 *E. coli* Rosetta strain (DE3: pLysS) (Novagen) was transformed with GST-Tev-VP4-His and grown at 37°C to an OD of 0.4 at 600 nm. NaCl (300 mM) was added to increase the osmolality of the nutrient medium. Simultaneous with the osmotic increase, the medium was supplied exogenously with 20 mM proline and the culture was induced with 1 mM IPTG for 4 hr at 30°C. Cells were centrifuged, resuspended in PBS (pH 7.4)/10 mM DTT with 200 µg/ml lysozyme and protease inhibitors, and rotated for 30 min at 37°C. Triton X-100 (1%) was added to the cells, which were then sonicated and the insoluble debris was sedimented by centrifugation for 20 min at 12,000× g. The clarified supernatant was then added to GST Sepharose 4B (Amersham Biosciences) matrix pre-equilibrated with PBS (pH 7.4)/10 mM DTT/1% Triton X-100. The matrix was washed three times with PBS (pH 7.4), and the protein was eluted with freshly prepared 10 mM reduced glutathione (in 50 mM Tris-HCl, pH 8.0). The eluate from GST Sepharose resin was further purified by adding the eluate to Ni-NTA His Bind resin (Novagen) pre-equilibrated with PBS (pH 7.4)/10 mM imidazole and additional 150 mM NaCl. The matrix was washed three times with PBS (pH 7.4)/50 mM imidazole, and the protein was eluted with 250 mM imidazole/PBS (pH 7.5). Protein purity was confirmed by SDS-PAGE and protein concentration was determined using a Bradford assay (Biorad). The expression and purification of GST-TEV-VP4ΔHD and GST-TEV-His were performed similarly.

### Hemolysis assay

Bovine RBCs were washed repeatedly in cold PBS immediately before use. Reactions (700 µl) were incubated at 37°C in hemolysis buffer (PBS, pH 7.4, 1 mM DTT and 200 ng BSA) with 0.5% RBCs, without or with 30 mM PEG (Fluka) and 10 µg/ml VP4, unless otherwise noted. Each time point for time course studies represented a separate reaction. End-point samples were removed after 30 min. Hemolysis was carried out in 10 mM Tris (pH 7.5)/150 mM NaCl containing 1 mM DTT and 200 ng BSA when the effect of ions was studied. Metal chlorides were added to the hemolysis reaction buffer at a final concentration of 40 mM. Reactions were centrifuged at 6,000× g for 5 min at 4°C to pellet unlysed cells. The absorbance of the supernatant was measured at 414 nm. Percentage of hemolysis was calculated as [(*A*_414_ (sample) − *A*_414_ (blank))/(*A*_414_ (water) − *A*_414_ (blank))] ×100. The blank reaction contained all components except protein and PEG. RBCs were hypotonically lysed by adding 50% water. Protease-treated RBCs were created by incubating cells at a concentration of 10% (v/v) in 10 mM Tris (pH 7.5)/150 mM NaCl, 20 mM MgCl_2_ containing 0.5 mg/ml proteinase K, 0.5 mg/ml trypsin, and 1.5 mM CaCl_2_ for 90 min at 37°C with gentle agitation. Proteases were inactivated with 2 mM PMSF and the cells were washed twice with cold 10 mM Tris (pH 7.5)/150 mM NaCl, 20 mM MgCl_2_ with 2 mM PMSF.

### RBC binding and integration

For determining RBC cell surface binding, hemolysis was carried out as described above at 37°C for 30 min. The cells were then centrifuged at 6,000× g for 5 min and the supernatant and pellet fractions were separated. Alkaline extractions were performed by resuspending the cell pellet in 700 µl of ice-cold 0.1 M Na_2_CO_3_ (pH 11.5), followed by a 30 min incubation on ice. The solution was layered on top of a 100 µl sucrose cushion, and the membrane-bound fraction was isolated by ultracentrifugation for 20 min at 65,000× g at 4°C. The membrane-bound pellet was resuspended in sample buffer, and the supernatant containing the peripherally associated proteins was TCA precipitated, washed with acetone, and resuspended in sample buffer for SDS-PAGE (13% acrylamide) and immunoblotting.

### Liposome preparation

LUVs or liposomes were generated using a Liposofast extruder (Avestin Inc., Ottawa, Canada) [24]. Chloroform solutions of lipids [PC (POPC, 1-palmitoyl-2-oleoyl-*sn*-glycero-3-phosphocholine); PE (POPE, 1-palmitoyl-2-oleoyl-*sn*-glycero-3-phosphoethanolamine); PS (POPS, 1-palmitoyl-2-oleoyl-*sn*-glycero-3-(phospho-L-serine); PG (POPG, 1-palmitoyl-2-oleoyl-*sn*-glycero-3-(Phospho-rac-1-glycerol); SM (sphingomyelin)] were mixed in the respective ratios and chloroform was evaporated at 37°C with a mild N_2_ flow. The lipid film was kept under vacuum for at least 3 h to eliminate traces of the organic solvent. To hydrate the lipid mixture, 0.25 ml of Hepes buffer saline (HBS, pH 7.5) was added to the dried phospholipid/sterol mixture (final total lipid concentration 10 mM), and the samples were incubated for 30 min at 37°C. The lipids were then resuspended by vortexing. The suspended lipid mixtures were frozen in liquid N_2_ and thawed at 37°C a total of five times to reduce the number of multilamellar liposomes and to enhance the trapped volumes of the vesicles [24]. Then the samples were passed at room temperature 21 times through the extruder equipped with a 100 nm pore size polycarbonate filter. The resulting liposomes were stored at 4°C and used within 2 weeks of production. Liposomes containing the fluorophore, Terbium-Dipicolinic acid [Tb(DPA)_3_^3-^], were prepared as above, except that HBS buffer included 3 mM TbCl_3_ (Alfa Aesar, Ward Hill, MA), and 9 mM 2,6-pyridinedicarboxylic acid (DPA, neutralized to pH 7). The resulting liposomes were separated from non-encapsulated [Tb(DPA)_3_^3-^] by gel filtration (Sepharose CL-6B-200, 0.7 cm inner diameter ×50 cm) in HBS buffer.

### Liposome permeabilization assay

Liposomes (100 µM total lipids) were suspended in 300 µl of buffer A (PBS, pH 7.4) containing 5 mM EDTA. The net initial emission intensity (F_0_) was determined after equilibration of the sample at 25°C for 5 min. Aliquots of 3 µl containing the amount of protein that gives the final concentration of 5 µg/ml were added and the samples were incubated for 30 min at 37°C. After re-equilibration at 25°C, the final net emission intensity (F_f_) of the sample was determined (i.e., after blank subtraction and dilution correction) and the fraction of marker quenched was estimated using (F_0_-F_f_)/(F_0_-F_T_), where F_T_ is the net emission intensity obtained when the same liposomes are treated with 3 mM Triton X-100 (i.e., under conditions of maximal release of the fluorophore).

### Steady state fluorescence spectroscopy

Intensity measurements were performed using the Fluorolog 3–21 spectrofluorimeter equipped with a 450 W xenon arc lamp, a double excitation monochromator, a single emission monochromator, and a cooled PMT. The excitation wavelength/bandpass, and the emission wavelength/bandpass were respectively: 278/2 and 544/4 nm for [Tb(DPA)_3_^3-^]. For [Tb(DPA)_3_^3-^] measurements, a 385 nm longpass filter was placed in the emission light path to block any second-order scatter emission light. Measurements were done in 4×4 mm quartz microcells stirred with a 2×2 mm magnetic bar as described previously [24].

### Liposome flotation assay

Binding reactions (75 µl) containing LUVs (400 µM) and protein (the amount of protein added was such that the ratio of LUVs to protein was same as that used in liposome permeabilization assay) were incubated at 37°C for 30 min. LUVs-bound and unbound proteins were separated by flotation through sucrose gradients, as liposomes float in the gradient when a g-force is applied, while free proteins sediment. Sucrose/HBS (225 µl of 67%) was added to the binding reactions and thoroughly mixed. The samples were overlaid with 360 µl of 40% sucrose, followed by 240 µl of 4% sucrose. Centrifugation was carried out for 50 min at 90,000× g at 4°C. Three 300 µl fractions (upper, middle, and bottom) were collected from the gradient. After trichloroacetic acid precipitation and resuspension in SDS sample buffer, samples were analyzed by SDS-PAGE and immunoblotting.

### Dynamic light scattering

The average size of the liposomes, before and after incubation with VP4, was determined by dynamic light scattering. Measurements were made at room temperature using a PDDLS Coolbatch 90T/PD2000DLS^Plus^ instrument (Precision Detectors, Inc., Franklin, MA) employing a 30-mW He-Ne laser source (658 nm) and a photodiode detector at an angle of 90°. VP4 and liposome concentrations were the same as that employed for the liposome permeabilization assays.

### Lactate dehydrogenase (LDH) release

The assay was carried out by plating Cos 7 cells (10,000 cells/well) and incubating the cells with 10 µg/ml protein for 30 min at 37°C. Aliquots of media (50 µl) were removed for the determination of LDH release. The CytoTox 96 cytotoxicity assay kit was used to determine the level of LDH released from the cells according to the manufacturer's instructions. After allowing 30 min of incubation with substrate, the A_490_ was determined using a BioTek Synergy 2 multi-mode microplate reader. As a control for total cell-associated LDH, Cos 7 cells in selected wells were lysed with 0.9% Triton X-100. Percentage LDH release was calculated by dividing the A_490_ released from samples by total cell-associated LDH release and multiplying by 100.

### Accession number

The GenBank (http://www.ncbi.nlm.nih.gov/Genbank/) accession number for SV40 VP4 is DAA06058.1.

## Supporting Information

Figure S1 **GST-VP4 size determination by SEC.** (A) The calibration plot for Superdex 200 10/30 GL column. Standard proteins used were bovine thyroglobulin (669 kD); horse spleen apoferritin (443 kD); sweet potato β-amylase (200 kD); yeast alcohol dehydrogenase (150 kD); bovine serum albumin (66 kD); ovalbumin (45 kD), and bovine carbonic anhydrase (29 kD). GST-VP4 is indicated by a solid square. Vo (determined using blue dextran) and Ve are the column void volume and the protein elution volume, respectively. (B) Chromatogram of the elution of purified GST-VP4 on the Superdex 200 10/30 GL column as visualized by monitoring absorbance at 280 nm.
(TIF)

Figure S2 **Substituting the hydrophobic domain of VP4 with transmembrane domain of leader peptidase abolishes VP4 lytic activity.** (A) GST-VP4, GST-VP4ΔHD, GST-VP4HD/LepTM1 (LepTM1), and GST-VP4HD/LepTM2 (LepTM2) were incubated with bovine RBCs for 30 min at 37°C. Released hemoglobin was measured by the A_414_ of the supernatant after centrifugation and the removal of unlysed cells. GST was used as a control. (B) [S^35^]-Met/Cys labeled VP4 binds GST-VP4. Radiolabeled VP4 was synthesized with reticulocyte lysate prior to GST-VP4 binding and isolation. VP4 was either synthesized alone (lanes 4-6) or from the VP2 transcript that supports the translation of VP2, VP3 and VP4 (lanes 1–3).
(TIF)

Text S1 The supporting text includes the supplemental materials and methods as well as supplemental references.
(DOC)
