# Supplementary figures and images for: The SV40 Late Protein VP4 Is a Viroporin that Forms Pores to Disrupt Membranes for Viral Release
Source: PLoS Pathog. 2011 Jun 30;7(6):e1002116. doi: 10.1371/journal.ppat.1002116 (PMC3128117; doi:10.1371/journal.ppat.1002116)

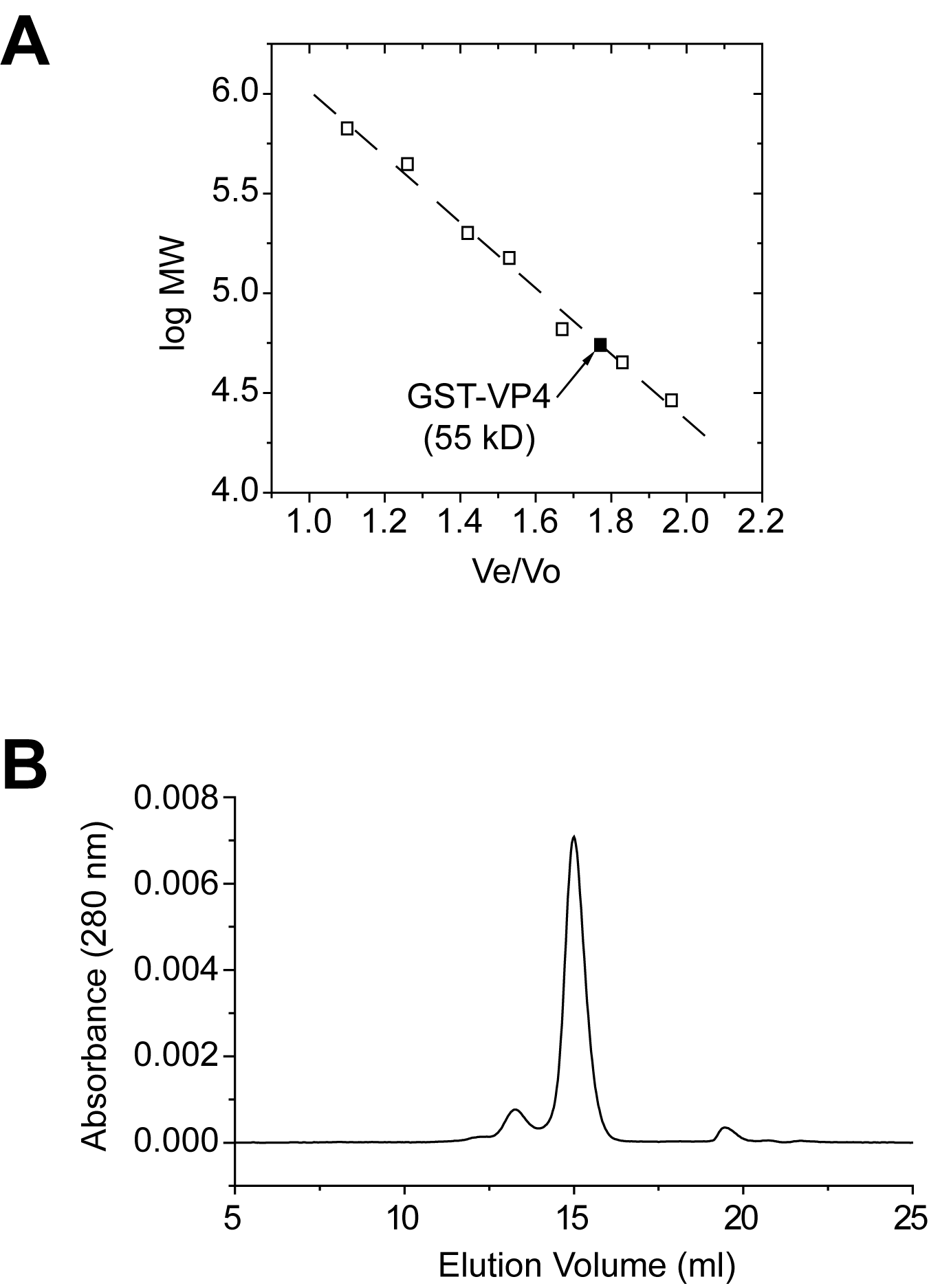

Supplement: Figure S1 — GST-VP4 size determination by SEC. (A) The calibration plot for Superdex 200 10/30 GL column. Standard proteins used were bovine thyroglobulin (669 kD); horse spleen apoferritin (443 kD); sweet potato β-amylase (200 kD); yeast alcohol dehydrogenase (150 kD); bovine serum albumin (66 kD); ovalbumin (45 kD), and bovine carbonic anhydrase (29 kD). GST-VP4 is indicated by a solid square. Vo (determined using blue dextran) and Ve are the column void volume and the protein elution volume, respectively. (B) Chromatogram of the elution of purified GST-VP4 on the Superdex 200 10/30 GL column as visualized by monitoring absorbance at 280 nm. (TIF) [file ppat.1002116.s001.tif]

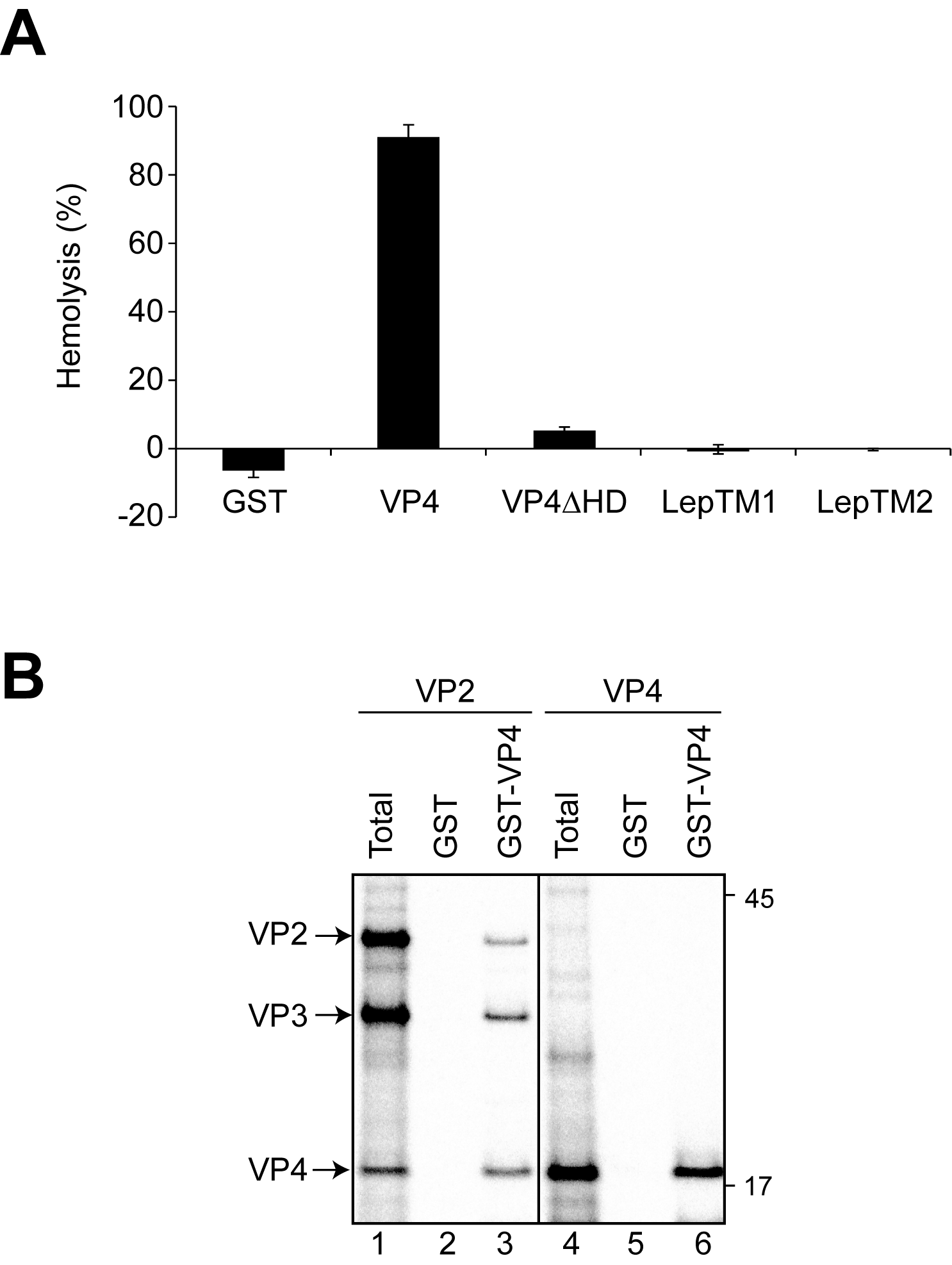

Supplement: Figure S2 — Substituting the hydrophobic domain of VP4 with transmembrane domain of leader peptidase abolishes VP4 lytic activity. (A) GST-VP4, GST-VP4ΔHD, GST-VP4HD/LepTM1 (LepTM1), and GST-VP4HD/LepTM2 (LepTM2) were incubated with bovine RBCs for 30 min at 37°C. Released hemoglobin was measured by the A414 of the supernatant after centrifugation and the removal of unlysed cells. GST was used as a control. (B) [S35]-Met/Cys labeled VP4 binds GST-VP4. Radiolabeled VP4 was synthesized with reticulocyte lysate prior to GST-VP4 binding and isolation. VP4 was either synthesized alone (lanes 4-6) or from the VP2 transcript that supports the translation of VP2, VP3 and VP4 (lanes 1–3). (TIF) [file ppat.1002116.s002.tif]
