# Supplementary material for: The SV40 Late Protein VP4 Is a Viroporin that Forms Pores to Disrupt Membranes for Viral Release
Source: PLoS Pathog. 2011 Jun 30;7(6):e1002116. doi: 10.1371/journal.ppat.1002116 (PMC3128117; doi:10.1371/journal.ppat.1002116)
Supplement: Text S1 — The supporting text includes the supplemental materials and methods as well as supplemental references. (DOC) [file ppat.1002116.s003.doc]

**SUPPORTING MATERIALS AND METHODS**

**Size exclusion chromatography**

Since GST was previously shown to dimerize in solution [1,2], the oligomeric state of GST-VP4 was determined by using size exclusion chromatography (SEC). GST-VP4 was resolved over a Superdex 200 10/30 GL column (GE Healthcare) using running buffer 20 mM Tris-HCl (pH 7.5), 300 mM NaCl. The column was calibrated using a variety of protein standards. GST-VP4 eluted with an average elution volume of 15.13 ml that corresponded to an estimated molecular weight of 55.0 kD. This indicated GST-VP4 was monomeric in solution. As a control, GST was analyzed under the same conditions. GST eluted with an average elution volume of 14.94 ml that corresponded to an estimated molecular weight of 60.0 kDa, in agreement with dimeric GST (data not shown).

**DNA constructs**

Since deletion of the hydrophobic domain abolished lytic activity it raised the question, can another transmembrane region substituted for the hydrophobic domain of VP4 support membrane disruption? To address this, the transmembrane domains of the model membrane protein leader peptidase (Lep) were substituted for the hydrophobic domain of VP4. Phusion site-directed mutagenesis was used according to the manufacturer’s recommendations to swap the hydrophobic domain of VP4 with transmembrane domains of leader peptidase (amino acids 4-22, MFALILVIATLVTGILWCV or amino acids 59-77, GWLETGASVFPVLAIVLIV) to create VP4 HD/LepTM1 or VP4 HD/LepTM2, respectively.

**In vitro translation and pull-down assay**

Binding of in vitro translated VP4 with GST-VP4 was tested by pull-down assay as previously described [3]. In brief, the late viral proteins were radiolabeled with [S^35^]-Meth/Cys by in vitro translation with rabbit reticulocyte lysate (Promega, Madison, WI). Reactions were divided into total lysate or added post-translation to freshly isolated GST-VP4 and GST bound to GSH-Sepharose in 0.5 ml of PBS (pH 7.4), 1.0% Triton X-100, and 5 mM DTT. As globin nonspecifically binds [S^35^]-Met/Cys and displays a similar mobility to VP4, it was removed from the total lysate sample by incubation in HBS with 1% NP-40 detergent and Ni-NTA His Bind resin (Novagen). Supernatant samples were precipitated with trichloroacetic acid and resuspended in SDS sample buffer. Samples were analyzed by tris-tricine reducing SDS-PAGE and developed by autoradiography.

**SUPPORTING REFERENCES**

1. Lim K, Ho JX, Keeling K, Gilliland GL, Ji X, et al. (1994) Three-dimensional structure of Schistosoma japonicum glutathione S-transferase fused with a six-amino acid conserved neutralizing epitope of gp41 from HIV. Protein Sci 3: 2233-2244.

2. Vargo MA, Nguyen L, Colman RF (2004) Subunit interface residues of glutathione S-transferase A1-1 that are important in the monomer-dimer equilibrium. Biochemistry 43: 3327-3335.

3. Daniels R, Sadowicz D, Hebert DN (2007) A Very Late Viral Protein Triggers the Lytic Release of SV40. PLoS Pathog 3: e98.
